# Development of an Enhanced High-Yield Influenza Vaccine Backbone in Embryonated Chicken Eggs

**DOI:** 10.3390/vaccines11081364

**Published:** 2023-08-15

**Authors:** Lizheng Guan, Jihui Ping, Tiago J. S. Lopes, Shufang Fan, Robert Presler, Gabriele Neumann, Yoshihiro Kawaoka

**Affiliations:** 1 Department of Pathobiological Sciences, School of Veterinary Medicine, Influenza Research Institute, University of Wisconsin-Madison, Madison, WI 53711, USAtiagojab@yahoo.com.br (T.J.S.L.); robert.presler@wisc.edu (R.P.); 2 Division of Virology, Department of Microbiology and Immunology, International Research Center for Infectious Diseases, The Institute of Medical Science, University of Tokyo, Tokyo 108-8639, Japan; 3 The Research Center for Global Viral Diseases, National Center for Global Health and Medicine Research Institute, Tokyo 162-8655, Japan

**Keywords:** vaccine, eggs, high yield, influenza A virus

## Abstract

Vaccination is an efficient approach to preventing influenza virus infections. Recently, we developed influenza A and B virus vaccine backbones that increased the yield of several vaccine viruses in Madin–Darby canine kidney (MDCK) and African green monkey kidney (Vero) cells. These vaccine backbones also increased viral replication in embryonated chicken eggs, which are the most frequently used platform for influenza vaccine manufacturing. In this study, to further increase the viral titers in embryonated chicken eggs, we introduced random mutations into the ‘internal genes’ (i.e., all influenza viral genes except those encoding the hemagglutinin and neuraminidase proteins) of the influenza A virus high-yield virus backbone we developed previously. The randomly mutated viruses were sequentially passaged in embryonated chicken eggs to select variants with increased replicative ability. We identified a candidate that conferred higher influenza virus growth than the high-yield parental virus backbone. Although the observed increases in virus growth may be considered small, they are highly relevant for vaccine manufacturers.

## 1. Introduction

In recent decades, influenza A viruses of the H1N1 and H3N2 subtypes, together with viruses representing the two lineages of influenza B viruses, have circulated in humans, although one of the influenza B virus lineages has not been detected since the start of the COVID-19 pandemic [1]. Annually, about 3 to 5 million cases of severe influenza virus infections and an estimated 290,000 to 650,000 deaths worldwide are attributed to influenza virus infections [2,3]. Moreover, influenza also imposes a considerable economic burden on society.

Various vaccines and antiviral drugs have been developed to diminish the consequences of influenza virus infection. To date, vaccination continues to be one of the best prevention strategies to reduce severe disease and fatalities associated with human influenza A and B viruses. The emergence of mutations in the major antigenic epitopes of hemagglutinin (HA) can render circulating viruses resistant to antibodies induced by an influenza virus infection or vaccination; therefore, vaccine strains must be frequently updated. Most egg-propagated influenza A vaccines are based on a vaccine virus backbone comprising six influenza viral RNA (vRNA) segments (i.e., all vRNA segments except for the HA and neuraminidase (NA) vRNAs) derived from the A/Puerto Rico/8/34 (PR8, H1N1) virus, a virus known to replicate efficiently. This vaccine virus backbone is then combined with the HA and NA vRNAs of the vaccine strain recommended by the WHO strain selection committee for the respective influenza season. Once the desired HA and NA vRNAs have been combined with the vaccine virus backbone, the resulting egg-propagated vaccine viruses are amplified in embryonated chicken eggs, which remain the most widely used platform for human influenza vaccine production (https://www.cdc.gov/prevent/cell-based.htm; accessed on 31 July 2023).

The yield of the vaccine virus is an important factor in vaccine manufacturing. Previously, we developed high-yield influenza A [4] and B [5] vaccine virus backbones by introducing random mutations into the internal genes. Sequential passages in MDCK cells resulted in high-yield vaccine virus backbones that also conferred efficient vaccine virus replication in embryonated chicken eggs. Here, we attempted to further improve the influenza A vaccine virus backbone for increased replication in embryonated chicken eggs.

## 2. Materials and Methods

### 2.1. Viruses and Cells

MDCK cells were grown in MEM containing 5% newborn calf serum (NCS); 293T human embryonic kidney cells were maintained in DMEM supplemented with 10% fetal bovine serum (FBS). Vaccine viruses IVR-186 (derived from A/Singapore/INFIMH-16-0019/2016, i.e., a seasonal clade 3C.2a1 H3N2 virus), NIB-112 (derived from A/Switzerland/8060/2017, i.e., a seasonal clade 3C.2a2 H3N2 virus), NYMC X-327 (derived from A/Kansas/14/2017, i.e., a seasonal clade 3C.3a1 H3N2 virus), and NYMC X-275 (derived from A/Michigan/45/2015, i.e., a clade 6B.1 pandemic 2009 H1N1 virus) were kindly provided by the National Institute for Biological Standards and Control (NIBSC; Potters Bar, UK). Other viruses in this study were generated by using reverse genetics.

### 2.2. Construction of Plasmids, Plasmid Libraries, and Virus Libraries

The HA and NA vRNA segments of the respective viruses were amplified by RT-PCR. The RT-PCR products were then inserted into the RNA polymerase Ι vector pHH21 [6]. To construct plasmid libraries, we introduced 1–4 random mutations into the six internal vRNA segments of the PR8-HY vaccine backbone by using error-prone PCR and the GeneMorph Ⅱ Random Mutagenesis Kit (Agilent, Santa Clara, CA, USA). The randomly mutated PCR products were then cloned into the pHH21 vector. The mutation rate of the generated plasmid libraries was confirmed by sequence analysis of individual bacterial colonies. All virus libraries were generated by using reverse genetics as described previously [6]. Supernatant (100 μL) derived from transfected 293T cells was inoculated into embryonated chicken eggs to generate virus stocks. The virus titers of the stocks were then measured by performing plaque assays in MDCK cells. Virus libraries were passaged in embryonated chicken eggs, as shown in Figure S1. Eggs were incubated for 48 h at 35 °C. After each passage, the hemagglutination (HA) titer was determined; based on the titer, 1000- or 10,000-fold dilutions of the virus libraries were used for the next passage in eggs.

### 2.3. Virus Amplification, Growth Kinetics, and Purification

Ten-day-old embryonated chicken eggs were inoculated with 2 × 10^3^ PFU of each virus. The allantoic fluids were collected 48 h later. The HA and virus titers of the allantoic fluids were determined in HA and plaque assays, respectively. To analyze the growth kinetics of viruses in embryonated chicken eggs, 2 × 10^2^ PFU of the virus were inoculated into 10-day-old embryonated chicken eggs. The allantoic fluids of three eggs were harvested at the indicated time points. HA and virus titers were determined in HA and plaque assays in MDCK cells, respectively.

For virus concentration and purification, viruses (2 × 10^2^ PFU) were grown in 10-day-old embryonated chicken eggs for 48 h. The allantoic fluids were then harvested and clarified by centrifugation at 3500 rpm at 4 °C for 30 min. The virus was precipitated from the clarified allantoic fluids (18,500 rpm, 2 h, 4 °C, Beckman Type 19 rotor), resuspended in 5 mL PBS, and purified by ultracentrifugation using a 30 mL linear 20–50% sucrose density gradient (20%, 30%, 35%, 40%, 45%, and 50%; 18,500 rpm, 2 h, 4 °C, Beckman Type 19 rotor). The virus band was collected from the sucrose gradient, diluted in PBS, and further centrifuged to remove sucrose (25,000 rpm, 2 h, 4 °C, Beckman SW32 rotor). This purified virus pellet was resuspended in 300 μL of PBS containing 0.1% β-propiolactone (BPL) at 4 °C overnight to inactivate the virus particles, followed by incubation at 37 °C for 45 min to inactivate the BPL. The final virus sample was aliquoted and stored at −80 °C. The total protein content of the virus concentrate was determined by utilizing the Pierce BCA protein assay kit (Thermo Scientific, Waltham, MA, USA) according to the manufacturer’s instructions.

Viral protein was deglycosylated using PNGase F (New England Biolabs, Ipswich, MA, USA). First, 2 μL of virus concentrate was denatured in a total volume of 10 μL according to the manufacturer’s instructions. Then, the sample was incubated at 37 °C for 1 h with 2 μL of a 1/8 dilution of PNGase F in the GlycoBuffer provided by the manufacturer and with NP-40 at a final concentration of 1%.

To assess the HA content, virus concentrate (2 μL) was mixed with water to a total volume of 20 μL. Twenty microliters of loading dye containing 2% (*v/v*) β-mercaptoethanol as a reducing agent were added to each protein sample. Samples were then heated to 95 °C for 5 min before loading onto a NuPage 4–12% Bris–Tris precast gel (Life technology, Carlsbad, CA, USA). Electrophoresis was carried out with 1× MOPS buffer (Bio-Rad, Hercules, CA, USA) at 150 V for 120 min, and the gel was then stained with SYPRO-Ruby (Sigma, St. Louis, MO, USA). The protein yield was quantified using Image J software (National Institutes of Health, New York, NY, USA). The amount of total protein is the sum of the amounts of HA1, HA2, NP, and M1; the HA amount is the sum of the amounts of HA1 and HA2. To calculate the HA content, we divided the HA amount by the amount of total protein and multiplied this value by the amount of total protein analyzed using gel electrophoresis.

### 2.4. Statistical Analysis

For the analysis of the HA and virus titers at a single time point, we used a one-way ANOVA followed by Tukey’s Post Hoc test. For the analysis of the viral total protein yield and HA content, statistical significance was determined by multiple comparisons using a one-way ANOVA test.

## 3. Results

### 3.1. Isolation of Enhanced High-Yield Vaccine Virus Backbones in Embryonated Chicken Eggs

**Figure 1:**
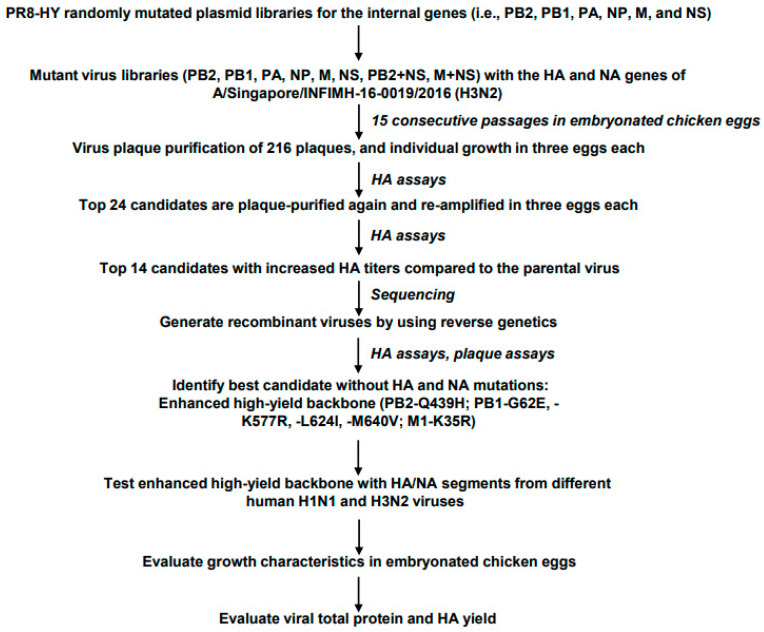
Flow chart summarizing the selection and testing of the enhanced high-yield vaccine backbone. See the text for details.

Previously, we developed a high-yield influenza A vaccine virus backbone that possesses amino acid changes in several viral proteins (i.e., in the PB2 polymerase protein (PB2-I504V), in the PB1 polymerase protein (PB1-M40L/G180W), in the PA polymerase protein (PA-R401K), in the nucleoprotein (NP-I116L), and in the nonstructural protein 1 (NS1-A30P/R118K)) and nucleotide changes in the promoter regions of three viral RNAs (i.e., PB2-C4U, PB1-C4U, and PA-C4U) [4]. Starting with the PR8-HY vaccine virus backbone we previously generated, we employed here a similar strategy to further improve the replication efficiency of the PR8-HY vaccine virus backbone in embryonated chicken eggs. First, we performed error-prone PCR to introduce one-to-four random amino acid changes, on average, into the viral proteins encoded by the internal viral RNA segments [4]. By using established reverse genetics approaches [6], we then used the mutated cDNA libraries to generate the following eight virus libraries (Figure 1 and Figure S1): six individual virus libraries possessing random mutations in each of the internal vRNAs (i.e., PB2, PB1, PA, NP, M (matrix), and NS (nonstructural)); one virus library possessing random mutations in the PB2 and NS vRNAs, selected because the PB2 and NS1 proteins (encoded by the NS vRNA) may affect viral growth [7,8]; and one library possessing random mutations in the M and NS vRNAs, because the M1 protein (encoded by the M vRNA) is associated with high-yield properties [9,10,11]. All eight virus libraries possessed the HA and NA vRNAs of A/Singapore/INFIMH-16-0019/2016 (H3N2; Singapore), i.e., a recent human H3N2 vaccine strain.

To select variants with enhanced high-yield properties, the eight individual virus libraries were passaged 15 times in embryonated chicken eggs (Figure 1 and Figure S1). In parallel, we combined aliquots of the eight individual virus libraries after the second passage to generate a ‘mixed library’, which was then subjected to 15 additional passages in embryonated chicken eggs (Figure S1). After the last passage, plaque assays were conducted in MDCK cells, and 20 virus plaques were randomly selected from each library, resulting in a total of 216 plaque-purified viruses. These viruses were each amplified in three embryonated chicken eggs, and then hemagglutination (HA) assays were performed. Twenty-four mutants exhibited HA titers that were at least two-fold higher than those of the parental PR8-HY virus (i.e., PR8-HY possessing the HA and NA vRNAs of Singapore). These enhanced high-yield candidates were again plaque-purified and amplified in three embryonated chicken eggs each. We collected samples at 36 h and 48 h post-infection to assess the HA titers and identified 14 viruses whose HA titers were at least two-fold higher than those of viruses bearing the parental PR8-HY backbone (Table S1).

Next, we sequenced the entire viral genomes of the top 14 enhanced high-yield candidates, most of which originated from the PB1 virus library (Table 1 and Table S1). Interestingly, most of the enhanced high-yield candidates from the PB1 virus library possessed PB2-Q439H and/or M1-K35R substitutions, suggesting that these changes emerged in the PB1 virus library. In addition, several of the enhanced high-yield candidates from the PB1 virus library also encoded PB1-G62E/K577R/L624I/M640V substitutions. Several other substitutions in the PB2, PB1, PA, M1, M2, and NS1 proteins were detected in subsets of the top 14 enhanced high-yield candidates. Although we did not intentionally introduce mutations into the HA and NA genes, several substitutions were detected in the HA and NA proteins (Table 1). In viruses derived from the PB1 virus library, the HA-K121E/T203I amino acid changes were detected frequently (amino acid numbers refer to mature H3 HA). For NA, the NA-I212T and V303I substitutions were detected frequently.

### 3.2. Identification of Mutation(s) That Increase Virus Replication in Embryonated Chicken Eggs

The top 14 enhanced high-yield candidates encode various combinations of amino acid changes in the internal viral proteins. To assess their relative contributions to increased virus replication, we generated recombinant viruses with different amino acid changes (RG-S3–RG-S16; Table 2). The parental PR8-HY virus encoding the HA and NA proteins of Singapore was generated and tested twice (RG-S1, RG-S2). To assess virus replication, three embryonated chicken eggs were each inoculated with an aliquot of the supernatant derived from plasmid-transfected 293T cells, and HA titers were measured 48 h later (Table 2). For one of the egg-amplified samples (i.e., the sample with the highest HA titer among the three replicates), we also performed plaque assays in MDCK cells (Table 2). Compared to the parental PR8-HY virus backbone (RG-S1, RG-S2), combinations of substitutions in the internal proteins increased virus titers by up to 4.3-fold (Table 2).

### 3.3. Selection of High-Yield Vaccine Virus Backbones for Additional Studies

**Figure 2:**
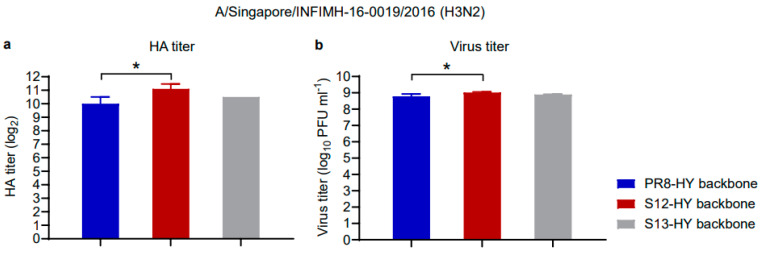
HA and virus titers in embryonated chicken eggs of two high-yield vaccine candidates. Viruses expressing the Singapore HA and NA proteins with the genetic backbone of the parental PR8-HY or the enhanced S12- and S13-HY candidates were inoculated into embryonated chicken eggs (2 × 10^3^ PFU/egg), and the HA (**a**) and virus (**b**) titers were measured 48 h later. Shown are the averages of three independently generated virus stocks. The titers were compared using a one-way ANOVA, followed by Tukey’s post-hoc test. * *p* < 0.05.

Based on the data shown in Table 2, we focused on the RG-S12 and RG-S13 enhanced high-yield candidates that showed the greatest increases in virus titers among the candidates with substitutions to their internal proteins (Table 2). After amplification in embryonated chicken eggs, the resulting virus stocks (named S12- and S13-HY, respectively) were then tested for their HA and virus titers in embryonated chicken eggs (Figure 2). For S12-HY (but not S13-HY), the HA and virus titers were significantly higher than for PR8-HY (*p* < 0.05).

### 3.4. Evaluation of Candidate Vaccine Viruses in Embryonated Chicken Eggs

**Figure 3:**
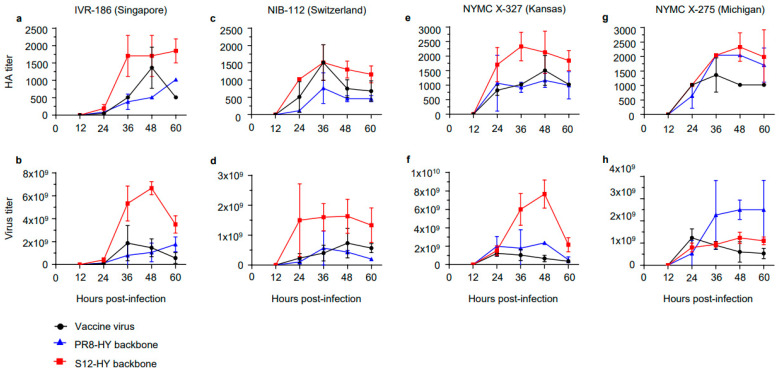
Growth curves in embryonated chicken eggs of vaccine viruses and recombinant viruses with different genetic backbones. The HA and NA genes of egg-grown Singapore (**a**,**b**), Switzerland (**c**,**d**), Kansas (**e**,**f**), and Michigan (**g**,**h**) vaccine viruses were combined with the genetic backbone of parental PR8-HY or enhanced S12-HY. For comparison, we also tested authentic egg-grown Singapore (IVR-186) (**a**,**b**), Switzerland (NIB-112) (**c**,**d**), Kansas (NYMC X-327) (**e**,**f**), and Michigan (NYMC-X275) (**g**,**h**) vaccine viruses from NIBSC. Ten-day-old embryonated chicken eggs were infected with 2 × 10^2^ PFU of the virus. The allantoic fluids of three eggs were harvested at the indicated time points. The HA and virus titers were measured by conducting HA and plaque assays in MDCK cells, respectively.

The enhanced high-yield vaccine backbone S12-HY was developed with the HA and NA vRNAs of the Singapore virus. To determine if this enhanced high-yield vaccine virus backbone also increased the titers of other vaccine viruses, we combined the S12-HY vaccine backbone with the HA and NA vRNAs of three other human influenza vaccine strains: A/Switzerland/8060/2017 (H3N2; Switzerland; clade 3C.2a2), A/Kansas/14/2017 (H3N2; Kansas; clade 3C.3a1), and A/Michigan/45/2015 (H1N1; Michigan; clade 6B.1) (Table S2). As controls, we tested the HA and NA vRNAs of the Switzerland, Kansas, and Michigan viruses in the parental PR8-HY backbone (Table S2). Compared to vaccine viruses (Figure 3, black lines), viruses with the enhanced high-yield (S12-HY) vaccine backbone (Figure 3, red lines) yielded HA titers that were up to four-fold higher (Figure 3a,c,e,f; Table S3). When comparing virus titers, the S12-HY backbone conferred a growth advantage to A/Singapore/INFIMH-16-0019/2016, A/Switzerland/8060/2017, and A/Kansas/14/2017, with increases of up to 11.5-fold for most time points tested (Figure 3b,d,f; Table S3). The >200-fold increase in virus titers for the A/Switzerland/8060/2017 HA/NA genes on the backbone of the S12-HY virus compared to the vaccine virus can be attributed to the low titers of the latter at 12 h after infection (Table S3). The S12-HY backbone did not increase the virus titers of A/Michigan/45/2015 (Figure 3h, Table S3).

### 3.5. Evaluation of Total Protein Yield and HA Content

**Figure 4:**
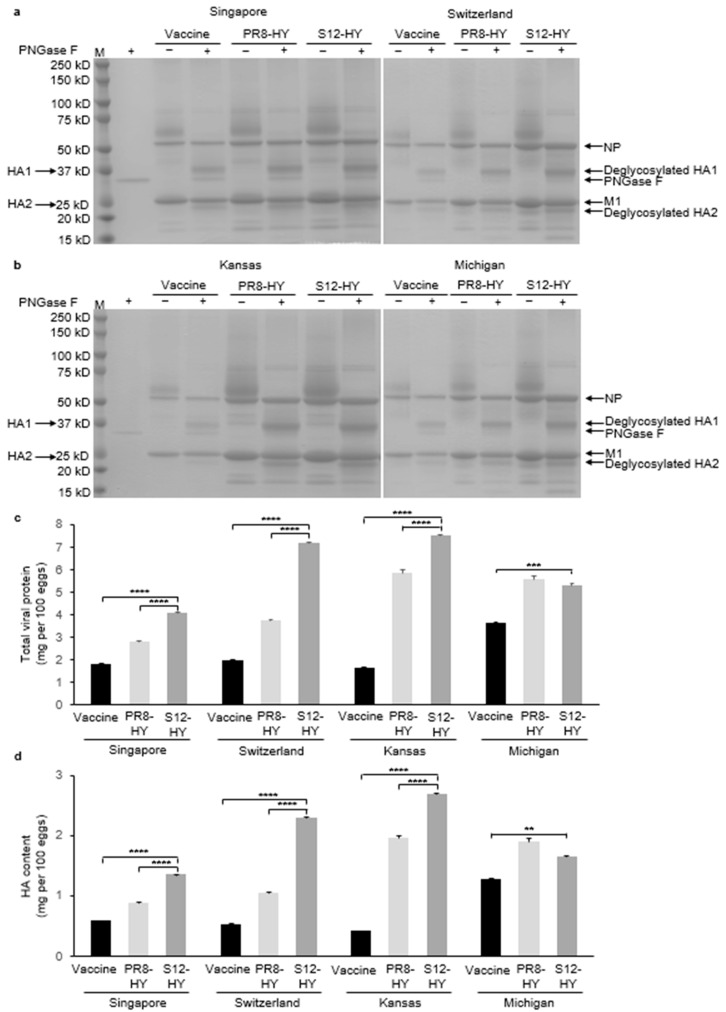
Total viral protein and HA content of vaccine viruses and recombinant viruses with different genetic backbones. Viral protein was deglycosylated using PNGase F (New England Biolabs) and then subjected to SDS-PAGE (**a**,**b**). HA contents (**d**) were calculated according to the amounts of total viral protein (**c**) and the relative amounts of HA). The HA contents are expressed in mg per 100 eggs. Asterisks denote a significant difference between groups. The values presented are the average of three independent values ± s.d. Statistical significance was determined by multiple comparisons using a one-way ANOVA test. ** *p* < 0.01; *** *p* < 0.001; **** *p* < 0.0001.

Total viral protein yield, and in particular the amount of HA, are important parameters for vaccine production. Therefore, we compared the total viral protein yield and HA content of our high-yield vaccine candidates propagated in embryonated chicken eggs. The S12-HY backbone yielded significantly more total viral protein (Figure 4b) and HA (Figure 4c) than the three H3N2 vaccine viruses (i.e., IVR-186 (Singapore), NIB-112 (Switzerland), and NYMC X-327 (Kansas)) and the control viruses based on the PR8-HY backbone (** *p* < 0.01, *** *p* < 0.001, **** *p* < 0.0001). For the H1N1 vaccine virus (i.e., NYMC X-275 (Michigan)), the S12-HY backbone yields significantly more total virus protein (Figure 4b) and HA (Figure 4c) than the vaccine virus, but not the virus based on the PR8-HY backbone. Collectively, these data establish that the S12-HY vaccine virus backbone developed here could increase the HA content of seasonal influenza vaccines, specifically those of the H3N2 subtype.

## 4. Discussion

Our enhanced high-yield vaccine virus backbone, S12-HY, significantly increased the HA content and viral titers of several seasonal influenza A vaccine viruses in embryonated chicken eggs compared to authentic vaccine viruses from NIBSC. Moreover, the enhanced high-yield vaccine virus backbone improved the HA content and viral growth properties relative to those of the parental high-yield PR8 backbone in some instances. Overall, the increases achieved with the S12-HY backbone may be considered small; however, a two-fold increase in titers and/or yield would double the number of vaccine doses that can be produced from the same number of eggs and is thus highly relevant for vaccine manufacturing, especially in times of high vaccine demand. Collectively, these data indicate that the enhanced high-yield vaccine virus backbone could be used in the future to increase the yield of seasonal influenza vaccines.

The enhanced high-yield vaccine virus backbone possesses six amino acid changes in three viral proteins compared to the parental PR8-HY vaccine backbone (i.e., PB2-Q439H, PB1-G62E/K577R/L624I/M640V, and M1-K35R). The PB2-Q439 residue is highly conserved among human, swine, and avian influenza A virus proteins; the histidine residue at this position of the enhanced S12-HY vaccine virus backbone has not been detected among circulating influenza viruses (Table S4). Similarly, the PB1-G62 and PB1-K577 residues are also highly conserved among human, swine, and avian influenza A virus proteins (Table S4). The PB1-L624 residue is highly conserved among human and swine influenza A viruses, whereas about 24% of avian influenza viruses encode an arginine at this position. Residue PB1-M640 (encoded by the parental PR8-HY vaccine backbone) is rarely found among human, swine, and avian influenza A viruses; interestingly, the valine residue selected from randomly mutated PB1 proteins is encoded by most human, swine, and avian influenza A viruses (Table S4). The M1-K35 residue is extremely common in nature, and the arginine residue detected in the S12-HY backbone has been found in a few circulating viruses (Table S4).

Amino acid 439 of PB2 is in the cap-binding domain of the PB2 protein, whereas residues 62 and 577/624/640 are in the fingers and thumb domains of the PB1 protein, respectively [12]. A previous study reported that PB1-577E enhances viral polymerase activity in human cells [13]. Whether the PB2 and PB1 substitutions identified in our study affect virus replication and polymerase activity remains to be studied. The M1 protein is an essential structural component of influenza viral particles and also plays a role in the regulation of vRNP nucleocytoplasmic shuttling [14]. Most viruses isolated in our study possessed the M1-K35R substitution after several passages in embryonated chicken eggs, suggesting selection for this substitution. The K35R substitution in the M1 protein increases M1 ubiquitination [15], but its role in viral replication remains unknown.

Traditionally, influenza vaccine viruses are generated by co-inoculating two influenza viruses into embryonated chicken eggs: the circulating virus against which the vaccine is supposed to confer protection and a virus that grows efficiently in embryonated chicken eggs, such as the PR8 virus. Antiserum against the efficiently replicating virus is then used to isolate reassortants that possess the HA and NA vRNA segments of the circulating virus in the genetic background of the PR8 virus. Several studies have found that the most frequently isolated gene constellations are 6:2 (i.e., the six ‘internal’ vRNA segments from PR8 and the HA and NA vRNA segments from the circulating virus) or 5:3 reassortants (with three vRNA segments derived from the circulating virus) [10,16,17]. The M vRNA segment almost always originates from PR8 [10,16,17] and has been shown to confer a higher yield [11]. Analysis of 5:3 reassortants has revealed that the PB1 vRNA segment is frequently derived from the circulating virus [10,16,17] by conferring a higher yield [18,19,20,21]. Thus, the high yield of a candidate vaccine virus is often conferred by the PR8 M vRNA segment and the PB1 vRNA segment of the circulating virus. Nonetheless, the yield of these reassortants is not always sufficient for large-scale vaccine production, and serial passages in embryonated chicken eggs may be needed to improve vaccine virus yield. Notably, the first candidate vaccine viruses for the 2009 H1N1 pandemic were of low yield, which had important consequences for global pandemic responses [22,23,24,25].

The low yield of the first generation of candidate vaccine viruses to the pandemic 2009 H1N1 viruses spurred multiple activities to increase yield by testing additional reassortants, multiple passages in embryonated chicken eggs or cultured cells, and the use of recombinantly generated candidate vaccine viruses [23,26,27,28,29,30,31,32,33]. Several mutations in HA (acquired through multiple passages in embryonated chicken eggs or cultured cells or introduced recombinantly) increased vaccine virus yield [26,27,28,29,30], but these mutations were strain-specific and may have affected antigenicity [29]. In our study, we also detected mutations in HA and NA, although these genes had not been targeted by random mutagenesis (see Table 1). In our study, we sought to develop a broadly applicable vaccine vector and therefore did not test these strain-specific HA substitutions in detail. Other recombinantly generated H1N1 candidate vaccine viruses of the 2009 pandemic possessed chimeric HA and/or NA proteins with extracellular domains derived from the circulating strain and transmembrane and/or intracellular domains derived from PR8 [34,35,36].

Serial passages in embryonated chicken eggs and cultured cells, as well as recombinant reverse genetics technologies, have also been used to optimize the yield of candidate vaccine viruses for highly pathogenic H5, H7H9, and H3N2v (H3N2 variant) viruses. Again, most of the yield-enhancing substitutions were detected in HA [37,38,39,40,41], but one study also demonstrated that an H5 candidate vaccine virus encoding a ‘full-length’ NA grew more efficiently than a virus encoding a neuraminidase protein with a deletion of 38 amino acids [42].

Few studies have attempted to specifically improve the PR8 backbone to develop a backbone that could increase the titers of various influenza viruses. Suzuki et al. [43] serially passaged PR8 virus in MDCK cells and detected seven amino acid substitutions in several viral proteins (PB2-D701N, PB1-H486N, PA-V44I, PA-G66D, NA-K247R, M1-A137T, NS2-S25L), none of which were detected in our study. Serial passages of PR8 in Vero cells resulted in several clonal lineages with different combinations of amino acid substitutions [44], which also did not overlap with the mutations isolated from our virus libraries. Based on the finding that reassortant candidate vaccine viruses often possess the PB1 vRNA segment of the circulating virus, Plant et al. [45] compared the PB1 amino acid sequence of PR8 with that of human influenza viruses. They then generated a ‘humanized’ PR8 PB1 protein encoding the G180E, S216G, S361R, Q621R, and N654S substitutions that promoted virus growth compared to a virus encoding the wild-type PR8 PB1 protein [45]. Interestingly, our first-generation high-yield PR8 vaccine virus backbone [4] also encoded a substitution at position PB1-180, although we detected tryptophan rather than alanine as detected by Plant et al. [45]. With serial passages in cultured cells and the introduction of selected amino acid substitutions, only a small number of possible amino acid substitutions can be tested. Therefore, we used random mutagenesis to create a large genetic diversity from which fast-growing variants are selected during virus replication. This approach identified yield-enhancing amino acid substitutions that were mostly different from those reported by others, indicating that multiple (combinations of) amino acid substitutions may enhance virus replication. Collectively, our study resulted in the creation of an enhanced vaccine virus backbone that could be used to improve the titers of seasonal influenza vaccine viruses.

## Figures and Tables

**Table 1:** Amino acid changes in the top 14 enhanced high-yield candidates.

Virus Isolate	Virus Library	HA Titer (2^n^) ^a^	Influenza Virus Protein							
			PB2	PB1	PA	M1	M2	NS1	HA (H3 Numbering ^b^)	NA
PR8-HY backbone	─	9.0~9.5	n/a	n/a	n/a	n/a	n/a	n/a	n/a	n/a
#34	Mixed	9.5~11.5	I105M	─	─	─	─	─	I140M	V303I
#12	PB2+NS	10.5~11.5	V504I	─	─	─	─	─	S124N	V303I
#3	M	9.5~11.5	─	K479M	─	─	─	─	I140M	D221N
#2	PB1	10.0~11.5	Q439H	─	─	K35R	R12K/R54P	─	K121E/T203I	V303I
#3	PB1	9.5~10.5	Q439H	L396I	─	K35R	─	─	K121E/T203I	I212T
#5	PB1	9.5~11.5	Q439H	G62E/K577R/ L624I/M640V	D294N	K35R	─	─	K121E/T203I	I212T
#8	PB1	10.5~11.0	Q439H	─	─	K35R	─	─	K121E/T203I	I212T
#9	PB1	10.5~11.5	Q439H	G62E/K577R/ L624I/M640V	─	─	─	─	K121E/T203I	V303I
#10	PB1	10.5~11.0	Q439H/ A624S	M655I	─	K35R	─	─	K121E/T203I	V303I
#11	PB1	9.5~11.5	Q439H	G62E/K577R/ L624I/M640V	─	K35R	─	─	I140M/V309I	V303I
#12	PB1	8.5~10.5	Q439H	─	─	K35R	─	E75G/ K78R	I140M	─
#14	PB1	10.5~11.5	Q439H	L396I/K479M	─	─	─		K121E/T203I	I212T
#16	PB1	10.5~11.5	─	G62E/T528N/ K577R/L624I/ M640V	─	─	R12K	─	K121E/T203I	V303I
#17	PB1	10.5~11.0	─	G62E/K577R/ L624I/M640V	R124K/ K339R	K35R	G16E	─	K121E/T203I	I212T

^a^ HA titers were taken from Table S1. ^b^ Numbers refer to the mature HA protein.

**Table 2:** Enhanced high-yield vaccine candidates of A/Singapore/INFIMH-16-0019/2016 (H3N2) with combinations of mutations identified in virus library screens.

Recombinant Virus	Amino Acid Changes Compared with PR8-HY Virus with the HA and NA Genes of A/Singapore/INFIMH-16-0019/2016					HA Titer (2^n^) ^b^			Viral Titer (PFU/mL) ^c^	Fold-Increase ^d^
	PB2	PB1	PA	M	NS					
RG-S1 ^a^	─	─	─	─	─	10.5 *	10.5	10.0	4.0 × 10^8^	
RG-S2 ^a^	─	─	─	─	─	10.0	10.0	10.5 *	4.2 × 10^8^	
RG-S3	I105M	─	─	─	─	9.5	10.5 *	9.5	7.0 × 10^8^	1.7
RG-S4	V504I	─	─	─	─	9.5	9.0	10.0 *	5.0 × 10^8^	1.2
RG-S5	─	K479 M	─	─	─	9.5	9.5	10.0 *	3.0 × 10^8^	<1.0
RG-S6	Q439H	─	─	K35R(M1)/R12K/R54P (M2)	─	9.5	10.5	10.5 *	8.0 × 10^8^	1.9
RG-S7	Q439H	L396I	─	K35R(M1)	─	9.5	11.0 *	10.5	8.0 × 10^8^	1.9
RG-S8	Q439H	G62E/K577R/ L624I/M640V	D294N	K35R(M1)	─	10.5 *	10.5	0	6.0 × 10^8^	1.4
RG-S9	Q439H	─	─	K35R(M1)	─	11.0 *	10.5	9.0	1.5 × 10^9^	3.6
RG-S10	Q439H	G62E/K577R/ L624I/M640V	─	─	─	9.5	10.0	10.5 *	9.0 × 10^8^	2.1
RG-S11	Q439H/ A624S	M655I	─	K35R(M1)	─	9.5	10.5 *	9.5	8.1 × 10^8^	1.9
RG-S12	Q439H	G62E/K577R/ L624I/M640V	─	K35R(M1)	─	11.0 *	10.5	10.5	1.8 × 10^9^	4.3
RG-S13	Q439H	─	─	K35R(M1)	E75G/K78R (NS1)	10.5	11.0	11.5 *	1.6 × 10^9^	3.9
RG-S14	Q439H	L396I/K479M	─	─	─	9.5	9.5	10.5 *	3.0 × 10^8^	<1.0
RG-S15	─	G62E/T528N/K577R/L624I/M640V	─	R12K(M2)	─	10.0 *	9.5	9.5	3.0 × 10^8^	<1.0
RG-S16	─	G62E/K577R/ L624I/M640V	R124K/ K339R	K35R(M1)/ G16E(M2)	─	9.5	9.5 *	9.5	5.0 × 10^8^	1.2

^a^ The control virus (possessing the internal genes of PR8-HY and the HA and NA genes of A/Singapore/INFIMH-16-0019/2016) was generated and titrated twice. ^b^ The HA titers were determined by performing HA assays with 0.5% turkey red blood cells. ^c^ The viral titers in MDCK cells were assessed for the allantoic fluids indicated by an asterisk. ^d^ Fold-increase was calculated by dividing the viral titer by the mean titer of the control viruses.

## Data Availability

All data associated with this study are available from the corresponding author upon reasonable request.

## Supplementary Materials

The following supporting information can be downloaded at: https://www.mdpi.com/article/10.3390/vaccines11081364/s1, Figure S1: Summary of the mutant virus libraries used and their consecutive passages in embryonated chicken eggs; Table S1: HA titers of high-yield candidates in embryonated chicken eggs at 36 h and 48 h post-infection; Table S2: Summary of viruses tested in this study; Table S3: Comparison of growth characteristics of S12-HY viruses possessing wild-type HA and NA genes; Table S4: Frequency in human, swine, and avian influenza A viruses of amino acid changes in the S12-HY vaccine virus backbone (influenza virus sequences are from https://www.ncbi.nlm.nih.gov/genomes/FLU/Database/nph-select.cgi?go=genomeset accessed on 8 July 2023).
