# Supplementary material for: Development of an Enhanced High-Yield Influenza Vaccine Backbone in Embryonated Chicken Eggs
Source: Vaccines (Basel). 2023 Aug 15;11(8):1364. doi: 10.3390/vaccines11081364 (PMC10459923; doi:10.3390/vaccines11081364)
Supplement: Supplementary file 1 [file vaccines-11-01364-s001.zip › vaccines-2509748-supplementary.pdf]

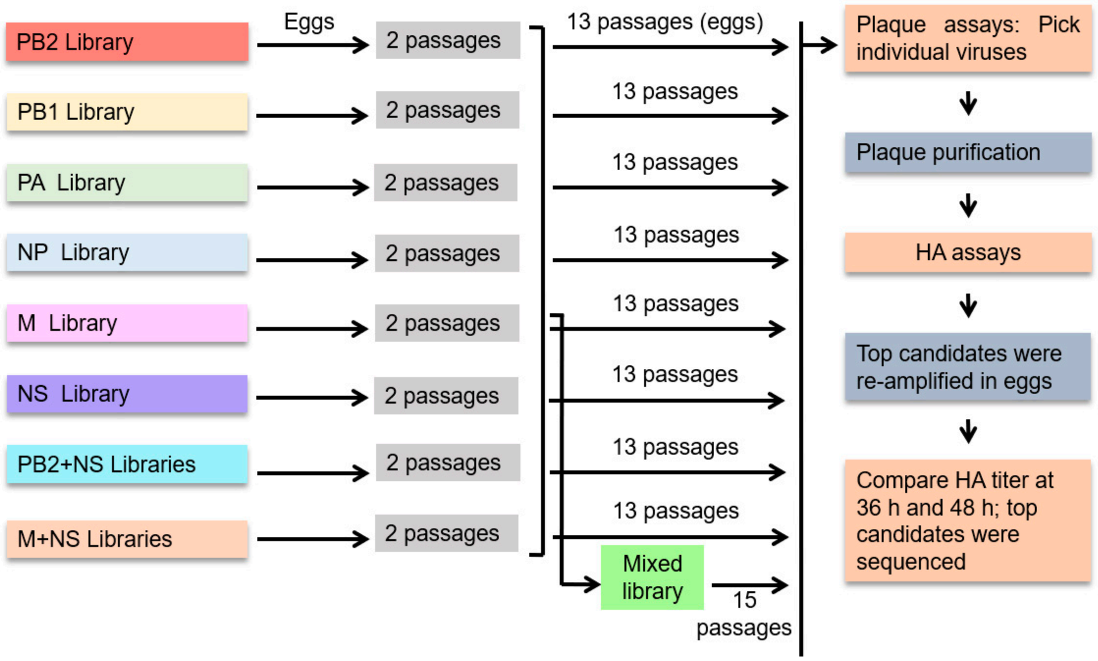

Figure S1. Summary of the mutant virus libraries used and their consecutive passages in embryonated chicken eggs.
All eight virus libraries were passaged twice in embryonated chicken eggs. Aliquots of the virus libraries were combined, resulting in a "mixed library". The individual libraries and mixed library were passaged 13 and 15 more times in embryonated chicken eggs, respectively, followed by virus plaque purification. In total, 216 plaques were picked, amplified in eggs, and their HA titers were measured. The candidates with the highest HA titers were re-amplified in eggs, and the viruses with increased HA titers were characterized in more detail.

Table S1. HA titers of high-yield candidates in embryonated chicken eggs at 36 h and 48 h post-infection

Virus library	Candidate no.[1]	HA titer at the indicated timepoint (log_2)									
		36 h post-infection					48 h post-infection				
		Egg 1	Egg 2	Egg 3	Average of HA titer	Fold-change[2]	Egg 1	Egg 2	Egg 3	Average of HA titer	Fold-change[2]
PR8-HY (Parental virus)		9.0	9.0	9.5	9.1	-	9.0	9.5	8.5	9.0	-
Mixed	14	9.5	10.5	10.0	10.0	1.8	10.0	10.0	10.5	10.1	2.1
	**34**	11.5	10.5	9.5	10.5	2.6	11.5	10.0	10.5	10.6	3.0
PB2+NS	3	11.5	10.0	10.0	10.5	2.6	9.5	10.0	10.0	9.8	1.7
	11	9.5	9.5	10.5	9.8	1.6	10.0	11.0	9.5	10.1	2.1
	**12**	10.5	10.5	10.5	10.5	2.6	10.5	11.5	11.0	11.0	4.0
M	**3**	11.5	9.5	11.0	10.6	2.8	10.5	10.5	10.5	10.5	2.8
NP	2	10.0	9.5	10.0	9.8	1.6	10.5	10.5	9.5	10.1	2.1
	8	10.0	9.5	10.0	9.8	1.6	9.5	9.5	10.0	9.6	1.5
PA	20	9.5	10.5	9.5	9.8	1.6	10.0	10.0	10.0	10.0	2.0
PB1	**2**	11.0	11.0	10.5	10.8	3.2	10.0	10.0	11.5	10.5	2.8
	**3**	10.5	10.5	9.5	10.1	2.0	10.5	10.5	10.5	10.5	2.8
	**5**	9.5	10.0	10.5	10.0	1.8	10.5	11.5	10.5	10.8	3.4
	7	11.0	9.5	10.5	10.1	2.0	10.5	9.5	10.5	10.1	2.1
	**8**	10.5	11.0	11.0	10.8	3.2	10.0	10.0	11.0	10.3	2.4
	**9**	10.5	11.5	11.5	11.1	4.0	11.5	10.5	10.5	10.8	3.4
	**10**	10.5	10.5	11.0	10.6	2.8	10.5	10.5	10.5	10.5	2.8
	**11**	9.5	10.5	10.5	10.1	2.0	11.5	11.5	10.5	10.8	3.4
	**12**	10.5	8.5	10.5	9.8	1.6	10.5	10.5	10.5	10.5	2.8
	13	10.5	12.0	9.5	10.6	2.8	10.0	10.0	10.0	10.0	2.0
	**14**	11.5	10.5	10.5	10.8	3.2	10.5	10.5	10.5	10.5	2.8
	15	9.5	10.5	10.5	10.1	2.0	10.5	9.5	11.0	10.3	2.4
	**16**	10.5	11.5	11.0	11.0	3.7	11.0	10.5	10.5	10.6	3.0
	**17**	11.0	11.0	11.0	11.0	3.7	10.5	10.5	10.5	10.5	2.8
	18	9.5	10.5	10.5	10.1	2.0	9.5	8.0	10.5	9.3	1.2

[1]The top 14 candidates are marked in bold-face type.

[2]Fold-change was calculated by dividing the average log_2 HA titer of the candidates by the average log_2 HA titer of the parental virus.

Table S2. Summary of viruses tested in this study

Original strain	Virus description	Subtype	Origin of viral genes				Passage history
			HA and NA	PB2	PB1	PA, NP, M and NS	
A/Singapore/INFIMH-16-0019/2016 (IVR-186)	Egg-grown vaccine virus	H3N2	A/Singapore/INFIMH-16-0019/2016	PR8	PR8	PR8	E8[a]
	Recombinant virus	H3N2	A/Singapore/INFIMH-16-0019/2016	PR8-HY*	PR8-HY	PR8-HY	E1
	Recombinant virus	H3N2	A/Singapore/INFIMH-16-0019/2016	S12-HY*	S12-HY	S12-HY	E1
A/Switzerland/8060/2017 (NIB-112)	Egg-grown vaccine virus	H3N2	A/Switzerland/8060/2017	PR8	PR8	PR8	E6[a]
	Recombinant virus	H3N2	A/Switzerland/8060/2017	PR8-HY	PR8-HY	PR8-HY	E1
	Recombinant virus	H3N2	A/Switzerland/8060/2017	S12-HY	S12-HY	S12-HY	E1
A/Kansas/14/2017 (NYMC X-327)	Egg-grown vaccine virus	H3N2	A/Kansas/14/2017	PR8	PR8	PR8	E17[a]
	Recombinant virus	H3N2	A/Kansas/14/2017	PR8-HY	PR8-HY	PR8-HY	E1
	Recombinant virus	H3N2	A/Kansas/14/2017	S12-HY	S12-HY	S12-HY	E1
A/Michigan/45/2015 (NYMC X-275)	Egg-grown vaccine virus	pdmH1N1	A/Michigan/45/2015	PR8	A/Michigan/45/2015	PR8	E11[a]
	Recombinant virus	pdmH1N1	A/Michigan/45/2015	PR8-HY	PR8-HY	PR8-HY	E1
	Recombinant virus	pdmH1N1	A/Michigan/45/2015	S12-HY	S12-HY	S12-HY	E1

*: High-yield backbones tested in this study.
[a]: Passage history of the vaccine viruses obtained from NIBSC.

Table S3. Comparison of growth characteristics of S12-HY viruses possessing wild-type HA and NA genes

		HA titer (log_2)					Virus titer (pfu/ml)				
Origin of HA and NA genes	Timepoint (h)	HA titer[a]			Fold-change[b]		Virus titer[a]			Fold-change[b]	
		Vaccine	PR8-HY	S12-HY	S12-HY/ Vaccine	S12-HY/ PR8-HY	Vaccine	PR8-HY	S12-HY	S12-HY/ Vaccine	S12-HY/ PR8-HY
A/Singapore/INFIMH-16-0019/2016 (H3N2)	12	ND[c]	ND	ND	-[d]	-	8.67E+03	2.67E+02	9.33E+03	1.1	35.0
	24	48	86	192	4.0	2.2	8.43E+07	1.36E+08	4.03E+08	4.8	3.0
	36	512	384	1,707	3.3	4.4	1.87E+09	8.00E+08	5.33E+09	2.9	6.7
	48	1,365	512	1,707	1.3	3.3	1.47E+09	1.07E+09	6.67E+09	4.5	6.3
	60	512	1,024	1,848	3.6	1.8	5.67E+08	1.77E+09	3.50E+09	6.2	2.0
A/Switzerland/8060/2017 (H3N2)	12	ND	ND	ND	-	-	7.00E+01	7.00E+01	1.47E+04	209.5	209.5
	24	512	116	1,024	2.0	8.9	2.33E+08	1.03E+08	1.50E+09	6.4	14.5
	36	1,507	768	1,507	1.0	2.0	4.00E+08	5.67E+08	1.60E+09	4.0	2.8
	48	753	462	1,307	1.7	2.8	7.33E+08	4.33E+08	1.63E+09	2.2	3.8
	60	683	462	1,165	1.7	2.5	5.67E+08	2.00E+08	1.33E+09	2.4	6.7
A/Kansas/14/2017 (H3N2)	12	ND	ND	ND	-	-	7.67E+05	3.80E+05	3.42E+05	0.4	0.9
	24	824	1,067	1,707	2.1	1.6	1.23E+09	2.00E+09	1.53E+09	1.2	0.8
	36	1,024	924	2,331	2.3	2.5	1.03E+09	1.78E+09	6.00E+09	5.8	3.4
	48	1,507	1,165	2,131	1.4	1.8	6.67E+08	2.37E+09	7.67E+09	11.5	3.2
	60	1,024	995	1,848	1.8	1.9	3.67E+08	5.27E+08	2.17E+09	5.9	4.1
A/Michigan/45/2015 (H3N2)	12	ND	ND	ND	-	-	2.00E+05	6.33E+03	1.20E+06	6.0	189.5
	24	1024	643	1024	1.0	1.6	1.23E+09	5.33E+08	8.00E+08	0.6	1.5
	36	1365	2048	2048	1.5	1.0	9.00E+08	2.27E+09	9.33E+08	1.0	0.4
	48	1024	2048	2331	2.3	1.1	6.00E+08	2.50E+09	1.23E+09	2.1	0.5
	60	1024	1707	1989	1.9	1.2	5.33E+08	2.50E+09	1.10E+09	2.1	0.4

[a]HA and virus titers are the averages of three individual samples at each timepoint.

[b]Fold-change is calculated by dividing the average values of HA and virus titers.

[c]Not detectable.

[d]Not calculated.

Table S4. Frequency in human, swine, and avian influenza A viruses of amino acid changes in the S12-HY vaccine virus backbone (influenza virus sequences are from https://www.ncbi.nlm.nih.gov/genomes/FLU/Database/nph-select.cgi?go=genomeset)

Viral protein	Position and mutation	Amino acid	Human H1N1 influenza viruses		Human H3N2 influenza viruses		Swine influenza viruses		Avian influenza viruses	
			Number of viruses	Frequency (%)	Number of viruses	Frequency (%)	Number of viruses	Frequency (%)	Number of viruses	Frequency (%)
PB2	Q439H	Q	14187	100.00%	20631	100.00%	7744	100.00%	19188	100.00%
		H	0	0	0	0	0	0	0	0
PB1	G62E	G	13767	99.93%	20510	99.70%	7579	99.65%	17792	98.65%
		E	1	<0.01%	40	0.19%	0	0	68	0.38%
		R	1	<0.01%	3	0.01%	8	0.11%	60	0.33%
		D	4	0.02%	0	0	0	0	0	0
		K	0	0	0	0	8	0.11%	114	0.63%
		Other (S/W)	1	<0.01%	4	0.02%	2	0.03%	0	0
	K577R	K	13735	99.70%	20514	99.76%	7484	98.40%	13452	74.59%
		R	20	0.14%	26	0.12%	90	1.18%	215	1.19%
		N	8	0.06%	5	0.02%	22	0.29%	11	0.06%
		Q	1	<0.01%	8	0.03%	2	0.03%	0	0
		E	7	0.05%	2	<0.01%	2	0.03%	17	0.09%
		M	1	<0.01%	0	0	1	0.01%	21	0.12%
		I	0	0	0	0	0	0	46	0.26%
		L	0	0	0	0	0	0	4260	23.62%
		Other (S/T)	4	0.03%	4	0.02%	3	0.04%	0	0
	L624I	L	8096	58.76%	20555	99.96%	7595	99.86%	13678	75.84%
		I	1	<0.01%	5	0.02%	9	0.04%	7	0.04%
		C	5676	41.19%	0	0	0	0	1	<0.01%
		R	0	0	0	0	0	0	4325	23.98%
		K	0	0	0	0	0	0	2	0.01%
		F	0	0	0	0	0	0	6	0.03%
		V	0	0	0	0	0	0	3	0.02%
		Other (Y/Q)	1	<0.01%	0	0	0	0	0	0
	M640V	M	15	0.11%	2	<0.01%	2	0.03%	12	0.07%
		V	8040	58.36%	20501	99.70%	7390	97.16%	13622	75.53%
		I	41	0.30%	56	0.27%	202	2.66%	35	0.19%
		L	1	<0.01%	3	0.01%	7	0.09%	13	0.07%
		S	1	<0.01%	0	0	0	0	4327	23.99%
		N	5678	41.21%	0	0	0	0	0	0
		A	0	0	0	0	3	0.04%	15	0.08%
		Other (D/F)	0	0	0	0	0	0	4	0.02%
M1	K35R	K	12637	99.99%	23837	99.98%	988	98.90%	20239	99.91%
		R	1	<0.01%	2	<0.01%	10	1.00%	17	0.08%
		N	0	0	0	0	1	0.10%	0	0
